# Interventions directed at men for preventing intimate partner violence: a systematic review protocol

**DOI:** 10.1186/s13643-021-01712-7

**Published:** 2021-06-01

**Authors:** Dina Idriss-Wheeler, Julia Hajjar, Sanni Yaya

**Affiliations:** 1 Interdisciplinary School of Health Sciences, University of Ottawa, 25 University Private, Ottawa, ON K1N 7K4 Canada; 2 School of International Development and Global Studies, University of Ottawa, 120 University Private, Ottawa, ON K1N 6N5 Canada; 3 The George Institute for Global Health, Imperial College London, London, UK

**Keywords:** Intimate partner violence, Interventions for men, Male perpetrators, Prevention, Abuse, Programme

## Abstract

**Background:**

Intimate partner violence (IPV) is a population health problem linked to a myriad of negative psychological, physical, emotional, sexual and reproductive health outcomes for women. The movement towards working with boys and men over the past couple of decades has increased the number of interventions specifically directed at men who perpetrate violence against a female partner. There is little evidence-based research on key characteristics of effective interventions directed at men to reduce or prevent IPV against female partners. The objective of this systematic review is to identify interventions specifically directed at males , as the perpetrators of violence against women, that have proven to be effective in preventing or reducing intimate partner violence.

**Methods:**

The following electronic databases will be used to search for peer-reviewed studies: MEDLINE (OVID), Embase (OVID), PsycInfo (OVID), CINAHL (EBSCO), Global Health (EBSCO), Gender Watch (ProQuest), Web of Science (Web of Knowledge), PROSPERO, Cochrane Central Register of Controlled Trials Database (Ovid) and SCOPUS. We will include randomized control trials, non-randomized studies of interventions published in peer-reviewed journals and relevant unpublished manuscripts, books/chapters and clinical or programme study reports. Studies have to demonstrate direction of effect (i.e. pre-post intervention/difference between groups) in terms of prevention or reduction in the outlined outcomes. Primary outcomes include change in behaviour and knowledge of male perpetrator regarding the impact of IPV on women as well as women’s experience of IPV. Secondary outcomes include change in behaviours around substance use and social activities, decrease in negative mental health outcomes and interactions with law enforcement. Studies will be screened, appraised and extracted by two reviewers; any conflicts will be resolved through discussion. Narrative synthesis will be used to analyse and present findings. If sufficient and comparable data is available, a meta-analysis will be conducted.

**Discussion:**

This review will provide synthesized evidence on interventions directed at males to reduce or prevent their perpetration of intimate partner violence against female partners. Implications for practice will include key characteristics of interventions proven to be effective based on evidence synthesis and certainty of findings. Recommendations for further research will also be considered.

**Systematic review registration:**

This protocol was submitted for registration in the International Prospective Register of Systematic Reviews (PROSPERO) on September 4, 2020.

**Supplementary Information:**

The online version contains supplementary material available at 10.1186/s13643-021-01712-7.

## Background

The WHO defines intimate partner violence (IPV) as ‘any behaviour within an intimate relationship that causes physical, psychological or sexual harm to those in the relationship’ [1]. This includes controlling behaviours that lead to isolation from family and friends, monitoring movements and restricting access to financial resources, employment, education or medical care [1]. Acknowledging that IPV affects both men and women, it remains that the latter are more severely impacted across all types of IPV [2–5].

As stated by the UN Women’s flagship report ‘Turning promises into action: Gender equality in the 2030 Agenda’, the 5th sustainable development goal (SDG) of gender equality matters across all the other SDGs calling for the elimination of all forms of violence against women and girls [6–8]. Nevertheless, 49 member states still lack laws protecting women against domestic violence [8]. Based on data from 87 countries, one in five women under the age of 50 will have experienced physical and/or sexual violence by an intimate partner within the last 12 months [7]. According to the Global Health Observatory data, on average, one in three ever partnered women around the world has experienced intimate partner violence in their lifetime [1].

Rates of violence increase in regions of conflict, particularly in low- and middle-income countries (LMICs) [9]. Syrian women refugees across the Middle East reported increased rates of domestic violence, sexual harassment and early or forced marriage [9]. Environmental disasters (i.e. drought, flooding, earthquakes, economic crisis, recession) have shown increased reports of violence against women with low levels of emergency preparedness to deal with this impact [10–12]. The recent actions imposed on citizens (i.e. social distancing, sheltering in place, restricting travel, closure of key community foundations) during COVID-19 have increased the risk of domestic violence, with reports around the globe of intimate partner homicide tied to stress and other factors related to the pandemic [13].

IPV is a population health problem linked to a multitude of poor health outcomes for women globally. Negative mental health outcomes include suicide, self-harm, depression, anxiety, post-traumatic stress disorder, as well as eating and sleep disorders [1]. Physical and behavioural repercussions are numerous and include injury, death, smoking and physical inactivity [1]. The sexual health and reproductive outcomes associated with IPV are dismal; they include unintended or unwanted pregnancy, abortion, unsafe abortion, sexually transmitted infections (including HIV), pregnancy complications, pelvic inflammatory disease, urinary tract infections and sexual dysfunction [1, 13–15]. In addition to the negative impacts on women’s morbidity and mortality, IPV has major economic implications for health, legal, police and social services [3, 16, 17].

Interventions to address IPV are geared towards both victims and perpetrators of IPV, usually through law enforcement or psychotherapeutic measures [18]. This work emerged through grassroots organizations and advocacy groups, service providers, researchers and policy makers who have developed shelters, advocated for change in laws and created indicator-based assessments of IPV during medical visits, as well as group counselling [2, 18]. Many of these approaches were to protect the abused individuals, the majority of whom were women [18]. Some criminal justice systems created specialized domestic violence courts as well as enacted legislation to increase protection for victims and children of victims [2]. The emerging innovative interventions continue to be rooted in law enforcement and/or psychotherapeutics, originating from North America and Western Europe, many of which focus on changing roles of race and gender [2, 18].

Several systematic reviews (registered on PROSPERO and/or the Cochrane Systematic Review Database) have assessed interventions that target domestic and/or intimate partner violence, with few focusing on male perpetrators. Some reviews include meta-analysis of the effectiveness of interventions to reduce intimate partner violence in general [19] or specifically in low- and middle-income countries which are geared at women [20]. Interestingly, Anderson, Meyer-Krause, Krause and Koss are completing a review that is investigating Web-based and mHealth interventions for domestic and intimate partner violence prevention [21]. Other systematic reviews are very specific in terms of screening women for IPV in healthcare settings [22] or preventing or reducing domestic violence perpetrated against pregnant women [23]. Fellmeth et al. [24] completed a systematic review on educational and skills-based interventions for preventing relationship and dating violence in adolescents and young adults. More recently, Rivas et al. [25] began to assess advocacy interventions for intimate partner abuse in women, looking at which interventions work for whom, why and in what circumstances.

Many of the systematic reviews of interventions to date have focused on screening for individuals who have experienced intimate partner violence, usually women, along with interventions to help women reduce or cope with their experience of IPV through education, advocacy, awareness or addressing social norms [26]. Over the past two decades, there has been a movement towards working with men and boys to reduce and/or prevent IPV through school-based initiatives, community mobilization or public awareness campaigns [27]. As stated in the 2007 WHO report on primary prevention of IPV and sexual violence:

to achieve and sustain large reductions in rates of intimate partner violence and sexual violence, social attitudes, norms and behaviours must be changed among men. Primary prevention strategies will NOT be effective if they focus on women and girls alone – *men and boys must be included*. Programmes working with men should approach men as partners and agents of change (p. 28) [27].

A 2007 systematic review specifically measured effectiveness of cognitive behavioural therapy (CBT) and programmes that include CBT on men’s physical abuse of their female partner [28]. Tarzia et al.’s [26] systematic review determined the effectiveness of interventions for male perpetrators and/or victims of IPV specifically in health settings. The review findings were predominantly related to high-income countries (Norway, UK, USA) [26]. Two interventions were in India and looked at reduction in alcohol/substance abuse and reported on increased identification and or referral of male patients who were either perpetrators or victims [26]. Although the review provided a knowledge gap specific to effective interventions for male perpetrators and victims in healthcare settings, it called for more research to further develop evidence base around effectiveness interventions directed specifically to men, particularly outside of alcohol-dependent populations. Tarzia et al. [26] concluded weak effectiveness of interventions for male perpetrators or victims of IPV in health-care settings. To our knowledge, no systematic review to date has looked at the effectiveness of interventions beyond healthcare settings, directed at men who perpetrated violence against female partners, with the aim of reducing or preventing intimate partner violence.

To address the gap in evidence, the objective of this systematic review is to identify interventions specifically directed a malest, as the perpetrators of violence against women, that have proven to be effective in preventing or reducing intimate partner violence. Evidence from existing interventions delivered through any modality across the globe will be included, characterized and analysed. Ultimately, the review findings will inform policy makers and non-government organizations on key characteristics of effective interventions and guide appropriate resource allocation. This work will contribute to understanding of evidence-based interventions that will reduce or prevent perpetration of IPV to reduce morbidity and mortality of women who are abused by their male partners.

### Research question for systematic review

What are the effects and effectiveness of available interventions targeting males in reducing or preventing intimate partner violence (IPV) perpetrated against their female partners?

## Methods

### Protocol registration and reporting

This systematic review will include quantitative studies of interventions (randomized and non-randomized designs) targeted at male perpetrators to prevent and/or reduce intimate partner violence against their female partner. The Preferred Reporting Items for Systematic Reviews and Meta-Analysis for Protocols (PRISMA-P) checklist was used to guide the development of this systematic review protocol [see Additional file 1] [29]. This protocol was submitted for registration in the International Prospective Register of Systematic Reviews (PROSPERO) on September 4, 2020.

### Inclusion criteria

#### Population

The population will be restricted to men who were part of an intervention aimed at reducing intimate partner violence against their female partners, globally. According to the WHO, females as young at 13 report experience of physical dating violence [30]. Therefore, this systematic review will capture interventions directed at males aged 13 or older who physically, sexually, emotionally and mentally abused their female partner. There will be no exclusions of study participants in terms of country or ethnicity. Relevant age as well as regional and national related information will be disaggregated and reported accordingly.

#### Types of studies to be included

This systematic review will include randomized and non-randomized studies (i.e. experimental, quasi-experimental, randomized trials, quasi-randomized trials) published in peer-reviewed journals. Observational studies that demonstrate the intervention’s findings (i.e. direction of effect measure; pre and post intervention findings) in terms of prevention or reduction in the outlined primary and secondary outcomes will be included. Studies that (i) do not have comparison or control group, (ii) only examine prevalence or predictors of factors associated with IPV perpetrated by men against women and (iii) cannot separate outcome data for female and male participants in a group/couples intervention will be excluded. Published letters, commentaries, conference abstracts and qualitative studies will not have details of primary and secondary outcome measurement effects and therefore will be excluded. To minimize risk of bias due to missing results, we will include unpublished manuscripts, books/chapters and grey literature (i.e. clinical or programme study reports).

#### Intervention

This review will include studies on interventions specifically designed for men and boys to prevent or reduce intimate partner violence against their female partner. Based on the WHO defined IPV, we will include interventions directed at the reduction of any or all of the following types of violence perpetrated by a man against his female partner: physical, sexual, emotional or mental. We will consider any type of intervention at the community, primary, secondary or tertiary level aimed at preventing or reducing intimate partner violence perpetrated by a male (aged 13 or older) against female partners. We will include interventions that are also directed at domestic violence (i.e. protecting both female partner and children in a household from violence). Interventions only directed at protecting children and not the female partner will be excluded. Types of intervention settings include, but are not limited to, the following: educational programmes, out-reach programmes, community health services, community mobilization, personal interventions, legal strategies, correctional and security and other [26, 29, 31]. The studies included should have quantitatively evaluated the effects of the interventions aimed at preventing or reducing perpetration of IPV. Studies will not be limited by geographical regions.

#### Comparison

This review will consider studies that compare the target (experimental/intervention) group to a comparison group (i.e. no intervention, other intervention, same group pre- and post-intervention). ‘No or other intervention’ includes a comparison group that does not receive a programme or may receive a standard/typical programme (i.e. educational pamphlet outlining information about IPV). Outcomes between intervention/experimental and comparison groups are then compared. Pre-post intervention group is a comparison of the same group receiving the intervention at different time points; effects are compared before and after intervention delivery in the same group.

#### Types of outcomes measures

Based on literature regarding interventions targeting men who have engaged in male-to-female violence (sexual, physical, emotional, mental) [21, 26, 31–36], this systematic review will report the following primary outcomes:

- Behaviour change in male perpetrator of IPV (i.e. direct measures of shifting attitudes, beliefs, norms or behaviours around violence, household roles or other relevant issues)
- Change in men’s knowledge about the impact of IPV on women
- Women’s experience of IPV from male partner after intervention (i.e. measures of reduction in IPV occurrences, improved mental health of female partner, other relevant measures showing change in women’s experience in IPV)

Secondary outcome examples will include:

- Change in male IPV perpetrator’s behaviour around substance use (reduction), mental health (improvement) and activities (social participation)
- Less security/police intervention order/instances against male IPV perpetrator

Measured effects will be reported based on statistically significant differences between experimental and comparison group on any of the primary and secondary outcomes outlined pre or post implementation of intervention.

### Information sources and search strategy

The following electronic databases will be used to search for peer-reviewed studies: MEDLINE (OVID), Embase (OVID), PsycInfo (OVID), CINAHL (EBSCO), Global Health (EBSCO), Gender Watch (ProQuest), Web of Science (Web of Knowledge), PROSPERO, Cochrane Central Register of Controlled Trials Database (Ovid) and SCOPUS. Studies will be identified using the medical subject headings (MeSH) and key terms based on previously published work in IPV interventions. The major concepts are ‘intervention’ AND ‘intimate partner violence’ AND ‘prevention’ AND ‘reduction’ [see Additional file 2 for example of MEDLINE’s search strategy]. The search will not be limited to men or male to ensure we are capturing all interventions (i.e. in case of interventions directed at couples). We will exclude interventions not directed at men during screening of abstracts. Search filters developed by the Scottish Intercollegiate Guidelines Network (SIGN) will be used to guide the search for randomized control trials (RCTs), observational studies and quasi-experimental studies [37]. To search for grey literature, books/chapters and programme reports, we will use Google, Google Scholar, the WHO website and Registers of Trials (i.e. ClincialTrials.gov). Studies published since January 2000 will be included. There will be no restrictions on language.

### Screening and selection process

Search results from the databases will be merged using the web-based software platform Covidence (Veritas Health Innovation) [38] and duplicate records of the same studies will be removed for the abstract screening phase. The titles and abstracts of each study in this screening phase will be independently examined by two reviewers (DIW and JH). Conflicts will be resolved through discussion; should the disagreement be due to difference in interpretation, a third reviewer (SY) will be asked to resolve the conflict. Irrelevant studies, based on the inclusion/exclusion criteria, will be removed and articles deemed relevant will move on to the full-text screening phase. Full-texts will be retrieved for all potentially relevant reports, repeating the same screening procedures—independent review by DIW and JH. Should there be a conflict or disagreement even following a discussion of a conflict, a third reviewer will once again be enlisted. Study authors will be contacted directly for clarification if necessary. Once the studies for full text have been decided upon, one reviewer (DIW) will manually review selected article reference lists to identify additional articles relevant to the review which will also undergo a full-text screening by the second reviewer. Reasons for exclusion will be decided upon by the review team and sufficiently documented. Final decisions on study inclusion will be confirmed by both reviewers. The selection process will be automated through Covidence and produce the necessary flow diagram outlining decision and reasons for exclusion.

### Assessment of risk of bias

Risk of bias assessment of individual study design characteristics will be completed using the Revised Cochrane risk-of-bias tool for randomized trials (RoB 2) and the Risk of Bias in non-randomized studies of Interventions (ROBINS-I) (i.e. quasi-experimental and observational) [see Additional file 3 for RoB 2 and ROBINS-I tool] [39, 40]. Two reviewers will assess all included studies for risk based using the outlined criteria to determine if the (i) randomized trial (RT) are at ‘low’, ‘high’ or ‘some concerns’ for risk of bias, and (ii) non-randomized studies of interventions (NRSIs) are at ‘low’, ‘moderate’, ‘serious’, ‘critical’ or ‘no information’ in terms of risk of bias. Reviewers will also determine the overall predicted direction of bias for each outcome (i.e. favours experimental, favours comparator, towards null, away from null, unpredictable or not applicable). Any disagreements and conflicts will be resolved through discussion, and if necessary, consult with a third reviewer.

### Data extraction

To minimize errors and reduce reviewer bias, two reviewers from complementary disciplines will independently extract the data for all included studies. Data extraction will be guided by the Cochrane Collaboration Data collection form Intervention review for RCTs and non-RCTs [41] [see Additional file 4]. The reviewers will pilot test the adapted form using three studies, compare their results and adjust coding instructions to ensure independent and harmonized extraction of the data. Categories to be extracted will include details of study method, population (intervention and control group), intervention description, sample size, study outcomes/results and any pertinent details (i.e. funding, ethics approvals, conflict of interest) deemed necessary to answer the review question. Extraction data will be compared to assure agreement, identify discrepancies and resolve conflicts through discussion between extractors. A third reviewer will be consulted if the conflict cannot be resolved. Original authors of the study will be contacted to retrieve missing or ambiguous data if deemed necessary.

### Data synthesis

Since the systematic review includes both randomized and non-randomized studies, we anticipate heterogeneity in both statistical analysis and methodology (i.e. diverse study designs, outcomes, contexts, populations and interventions). If sufficient and comparable data is available, a meta-analysis will be conducted using STATA version 16.0 to generate individual study effect estimates and a pooled estimate summarizing effectiveness. The anticipated heterogeneity of the studies estimating different yet related intervention effects will require a random-effects method, using an inverse-variance approach, for meta-analysis. If included studies do not yield effects which can be pooled, we will instead conduct a narrative synthesis as described by Popay et al. [42]. Furthermore, the ‘Synthesis without meta-analysis’ (SWiM) in systematic reviews guidelines, nine-item checklist, will be used to promote transparent reporting of reviews of interventions since meta-analysis was not be possible [43].

The narrative synthesis will allow us to explore four main elements: (i) a theoretical model of the interventions work, why and for whom; (ii) a preliminary synthesis to organize findings from the included studies (i.e. direction of effects and effect sizes); (iii) explore relationships in the data to consider factors that might explain any differences in direction and size effects across the included studies; and (iv) assess the robustness of the synthesis product (i.e. strength of evidence to draw conclusions about likely size and direction of effect, generalize on effect size to heterogenous groups/contexts) [42]. SWiM’s reporting items will complement the narrative synthesis and will be used as the extension to PRISMA. We will synthesize key elements of the *methods* (i.e. description of grouping of studies for synthesis; description of standardized metric methods use, description of synthesis methods, criteria used to prioritize results for summary and synthesis, investigation of heterogeneity in reported effects, certainty of evidence, data presentation methods), *results* (i.e. description of the synthesized findings and certainty of findings for each comparison outcome) and *discussion* (i.e. reporting the limitation of the synthesis methods used and or the grouping used in the synthesis and how they affect the conclusions in relation to effectiveness of the interventions designed to reduce or prevent IPV perpetrated by men against their female partners) [43]. Where possible, and depending on included studies, we will determine the best possible statistical synthesis (i.e. summarizing effect estimates, combining *p* values, vote counting based on direction of effect). We will perform a sub-group analysis on different regions to consider outcomes and characteristics from low- and middle-income countries and those from high income countries. We will also conduct any relevant analysis related to age of participants.

### Assessing certainty of evidence

We will use the Grades for Recommendation, Assessment, Development and Evaluation (GRADE) approach to assess the certainty of the body of evidence following guidelines described by Schünemann et al. [44]. The GRADE approach will consider within and across study risk of bias (ROB), inconsistency, indirectness of evidence, imprecision of the effect estimates and risk of publication bias for each individual outcome and these will be presented in the ‘summary of findings’ table [45]. There are four levels of certainty (high, moderate, low, very low) and the starting point for rating and certainty of evidence will be categorized into either randomized trials (RT) or non-randomized study of interventions (NRSI). RTs or studies evaluated with Risk of Bias In non-randomized Studies of Interventions (ROBINS-I) tool start as high certainty of evidence while NRSI as low certainty evidence [40, 45]. Two review authors will, independently, use the five reasons for lowering (i.e. ROB, inconsistency, indirectness, imprecision, publication bias) or raising certainty in case of NRSI (i.e. large effect, dose response, plausible confounding) [39, 45]. Conflicts will be resolved through discussion and/or input from additional reviewer. The grading of the certainty of evidence will be reported in the results section for each outcome discussing rationale for downgrading or upgrading the evidence as seen in the summary of findings table.

## Discussion

This review will reveal findings from synthesized evidence on interventions directed at males to reduce or prevent their perpetration of intimate partner violence against their female partners. The systematic review will aim to provide information on all important outcomes (both beneficial and adverse outcomes), discuss potential biases in the review process, outline applicability of the evidence, analyse the certainty of evidence for the outcomes and discuss agreements or disagreements with previous research [44]. Implications for practice and research will be considered, keeping in mind a broad international perspective so that recommendations regarding future interventions could be applied in diverse settings.

One consideration in performing this systematic review is the decision to only include interventions directed at male perpetrators of IPV against their female partners. Although we are only including interventions directed at males, the search strategy will not filter out interventions based on ‘men’ or ‘male’ from the title or abstract as they may not always provide this detail. Instead, this will be completed during the abstract screening process to ensure relevant interventions are not missed. It is possible that interventions directed at preventing or reducing IPV maybe combined or delivered with another intervention not related to IPV; if the study does not provide disaggregated results related to outcomes of an intervention to reduce or prevent IPV by the man towards his female partner, the intervention will be excluded. Some of the interventions may address one type of IPV (i.e. physical, emotional, psychological, sexual); we will include studies that provide effect sizes regarding outcomes on interventions directed at reducing or preventing any one or all forms of IPV.

The ethical and safety considerations for violence against women intervention research outlines specific recommendations regarding confidentiality of families, appropriate training for research within this context, and a minimum standard of care for comparison groups [46]. Given the nature of the intervention (prevention or reduction of IPV), we may not find relevant experimental and quasi-experimental studies of interventions with large effect sizes [47]. Observational (cross-sectional or longitudinal) studies assessing an intervention effect of a prevention/reduction of IPV programme will most likely yield low certainty of evidence (i.e. issue of confounding factors).

Any changes to the protocol while conducting the systematic review will be updated and reported in PROSPERO as well as the final manuscript which is intended for publication.

## Supplementary Information

**Additional file 1.** PRISMA-P 2015 Checklist.

**Additional file 2.** Example Search Strategy.

**Additional file 3.** The Risk of Bias in Non-randomized Studies – of Interventions (ROBINS-I) assessment tool.

**Additional file 4.** Data collection form.

## Data Availability

Not applicable; the protocol does not contain any data.

## Abbreviations

COVID-19: Coronavirus disease of 2019
GRADE: Grades for Recommendation, Assessment, Development and Evaluation
IPV: Intimate partner violence
JBI: Joanna Briggs Institute
LMICs: Low- and middle-income countries
NRSI: Non-randomized studies of interventions
PRISMA–P: Preferred Reporting Items for Systematic Reviews and Meta-Analysis for Protocols
PROSPERO: International Prospective Register of Systematic Reviews
RCT: Randomized control trial
ROB: Risk of bias
ROBINS-I: Risk of Bias In non-randomized Studies of Interventions
RT: Randomized trial
SDG: Sustainable development goals
SIGNS: Scottish Intercollegiate Guidelines Network
SWiM: Synthesis without meta-analysis
UN: United Nations
WHO: World Health Organization
