# Supplementary material for: Interventions directed at men for preventing intimate partner violence: a systematic review protocol
Source: Syst Rev. 2021 Jun 1;10:161. doi: 10.1186/s13643-021-01712-7 (PMC8166527; doi:10.1186/s13643-021-01712-7)
Supplement: Supplementary file 2 — Additional file 2. Example Search Strategy. [file 13643_2021_1712_MOESM2_ESM.docx]

**Additional File 2 – Example Search Strategy**

| Ovid MEDLINE® Search ALL 1946 to August 28^th^, 2020; Performed March 28^th,^ 2020 |
| --- |
| 1. exp Intimate Partner Violence/ |
| 2. ((sex* or physical or psychological or mental or emotion*) adj1 (abuse or violence)).ti,ab,kf. |
| 3. ((sex* or physical or psychological or mental or emotion*) adj2 (abuse or violence)).ti,ab,kf. |
| 4. (rape* or raping).ti,ab,kf. |
| 5. exp Battered Women/ |
| 6. exp Gender Based Violence/ |
| 7. exp Domestic Violence/ |
| 8. domestic abuse.ti,ab,kf. |
| 9. exp Spouse Abuse/ |
| 10. exp Rape/ |
| 11. sexual abuse/ |
| 12. exp Physical Abuse/ |
| 13. 1 or 2 or 3 or 4 or 5 or 6 or 7 or 8 or 9 or 10 or 11 or 12 |
| 14. Education/ or Curriculum/ or Program/ or Intervention/ or Treatment/ or Counselling/ or Instruction/ |
| 15. Awareness/ |
| 16. Program Evaluation/ or Program Development/ |
| 17. ((educat* or training or counsel* or learn* or teach* or instruction* or curricul* or syllab* or awareness) adj2 (Intervention* or Program*)).ti,ab,kf. |
| 18. ((educat* or awareness) adj2 campaign?).ti,ab,kf. |
| 19. ((classroom* or school* or peer* or communit*) adj3 (Intervention* or Program*)).ti,ab,kf. |
| 20. ((social media* or mhealth or mlearning or mobile health or mobile learning or facebook* or twitter* or instagram* or YouTube or "You Tube" or Google Hangout* or web* or digital* or internet* or mobile app* or app or apps or blog* or vlog* or weblog* or web-log*) adj3 (program* or intervention* or campaign?)).ti,ab,kf. |
| 21. ((interactive or computer-assist*) adj3 (tutorial* or program* or intervention* or instruction*)).ti,ab,kf. |
| 22. ((education* or instruction* or awareness) adj3 (tutorial* or video* or webcast* or vlog* or blog* or mobile app* or app or apps)).ti,ab,kf. |
| 23. 14 or 15 or 16 or 17 or 18 or 19 or 20 or 21 or 22 |
| 24. exp clinical studies as topic/ or Random Allocation/ or Double Blind Method/ or Single Blind Method/ or clinical trial/ or PLACEBOS/ |
| 25. (clinical study or clinical trial, phase i or clinical trial, phase ii or clinical trial, phase iii or clinical trial, phase iv or controlled clinical trial or randomized controlled trial or multicenter study or clinical trial).pt. |
| 26. ((clinical adj trial$) or controlled trial*).tw. |
| 27. ((singl$ or doubl$ or treb$ or tripl$) adj (blind$3 or mask$3)).tw. |
| 28. placebo$.tw. |
| 29. (allocated adj2 random$).tw. |
| 30. Epidemiologic studies/ or exp case control studies/ or exp cohort studies/ or Cross-sectional studies/ or controlled before-after studies/ or interrupted time series analysis/ |
| 31. (comparative study or evaluation study or observational study).pt. |
| 32. (Case control or (before adj1 after) or interrupted time serie* or (pre* adj2 post*)).tw. |
| 33. (cohort adj (study or studies)).tw. |
| 34. Cohort analy$.tw. |
| 35. (Follow up adj (study or studies)).tw. |
| 36. (observational adj (study or studies)).tw. |
| 37. (Longitudinal or quasi-experimental* or quasiexperimental* or Retrospective or Nonequivalent Group* or Regression discontinuity or Instrumental variable or Natural experiment*).tw. |
| 38. Cross sectional.tw. |
| 39. clinical study reports.af. |
| 40. case reports.af. |
| 41. 24 or 25 or 26 or 27 or 28 or 29 or 30 or 31 or 32 or 33 or 34 or 35 or 36 or 37 or 38 or 39 or 40 |
| 42. exp animals/ not humans.sh. |
| 43. 13 and 23 and 41 |
| 44. 43 not 42 |
| 45. limit 44 to yr="2000-current" |
